# A novel murine model of atrial fibrillation by diphtheria toxin-induced injury

**DOI:** 10.3389/fphys.2022.977735

**Published:** 2022-10-31

**Authors:** Theresa Trieu, Philbert Mach, Kaitlyn Bunn, Vincent Huang, Jamie Huang, Christine Chow, Haruko Nakano, Viviana M. Fajardo, Marlin Touma, Shuxun Ren, Yibin Wang, Atsushi Nakano

**Affiliations:** ^1^ Department of Molecular, Cell, Developmental Biology, School of Life Science, University of California, Los Angeles, Los Angeles, CA, United States; ^2^ Department of Pediatrics, David Geffen School of Medicine, University of California, Los Angeles, Los Angeles, CA, United States; ^3^ Departments of Anesthesiology, Physiology, David Geffen School of Medicine, University of California, Los Angeles, Los Angeles, CA, United States; ^4^ Department of Medicine, David Geffen School of Medicine, University of California, Los Angeles, Los Angeles, CA, United States; ^5^ Department of Cell Physiology, The Jikei University School of Medicine, Tokyo, Japan

**Keywords:** atrial fibrillation, diphtheria toxin, non-genetic cause, amiodarone, sarcolipin (SLN)

## Abstract

The treatment of atrial fibrillation (AF) continues to be a significant clinical challenge. While genome-wide association studies (GWAS) are beginning to identify AF susceptibility genes (Gudbjartsson et al., Nature, 2007, 448, 353–357; Choi et al., Circ. Res., 2020, 126, 200–209; van Ouwerkerk et al., Circ. Res., 2022, 127, 229–243), non-genetic risk factors including physical, chemical, and biological environments remain the major contributors to the development of AF. However, little is known regarding how non-genetic risk factors promote the pathogenesis of AF (Weiss et al., Heart Rhythm, 2016, 13, 1868–1877; Chakraborty et al., Heart Rhythm, 2020, 17, 1,398–1,404; Nattel et al., Circ. Res., 2020, 127, 51–72). This is, in part, due to the lack of a robust and reliable animal model induced by non-genetic factors. The currently available models using rapid pacing protocols fail to generate a stable AF phenotype in rodent models, often requiring additional genetic modifications that introduce potential sources of bias (Schüttler et al., Circ. Res., 2020, 127, 91–110). Here, we report a novel murine model of AF using an inducible and tissue-specific activation of diphtheria toxin (DT)-mediated cellular injury system. By the tissue-specific and inducible expression of human HB-EGF in atrial myocytes, we developed a reliable, robust and scalable murine model of AF that is triggered by a non-genetic inducer without the need for AF susceptibility gene mutations.

## Introduction

Atrial fibrillation (AF) is the most common arrhythmia that leads to congestive heart failure, thromboembolism, and bleeding related to the anticoagulant therapy, and contributes significantly to the morbidity and mortality (Lip et al., 2012; Wilke et al., 2013; Andrade et al., 2014). AF-related hospitalizations (primary or secondary diagnosis) show an upward trend since 1996 (Coyne et al., 2006; Ball et al., 2013; Patel et al., 2014). Despite the numerous therapeutic modalities currently available, treatment of AF continues to be a significant clinical challenge (Kirchhof et al., 2009; January et al., 2014a; January et al., 2014b). This is largely due to the incomplete understanding of the disease mechanism of AF. Multiple pathomechanisms are proposed to explain the initiation and progression of AF including ectopic automaticity, reentrant electric circuits and atrial remodeling. Many of these insights into the mechanism of AF are developed from studies in both large and small animal models (Nishida et al., 2010). Large animals are physiologically closer to humans and have provided electrophysiological insights. However, the limitations of large animal models are that the genetic analyses are hard to perform and relatively small number of animals can be examined at once. Small animals including mutant mouse models have provided insight into the genetic mechanism of AF. However, each genetic mutation elucidates at best a very small and specific component of the broad spectrum of AF, and the induction of AF is currently inefficient, time-consuming and uncontrollable. Therefore, animal models have not been able to fully contribute to identifying the molecular mechanism of AF to date.

Using highly specific and sensitive atrial Cre (causes recombination) knockin line (Sln-Cre) and diphtheria toxin (DT)-induced cell damage, we propose to establish a new genetically unbiased mouse model of AF. Our preliminary data suggest that more than 60% of the mice developed AF within 2 months with simple intraperitoneal (i.p.) injection of DT. Unlike previously published mouse lines and methods, this model induces genetically unbiased atrial damage, does not require transesophageal rapid pacing, and demonstrates high frequency of AF induction in a relatively short period of time. Furthermore, we can control the severity of atrial damage by modulating the dosage and timing of DT injection. Our AF model is 1) easy, 2) quick, 3) efficient, 4) controllable, 5) scalable and 6) genetically unbiased. With these six advantages, we hope that this model will change the approach to studying AF.

## Methods

### Mouse lines

**FIGURE 1:**
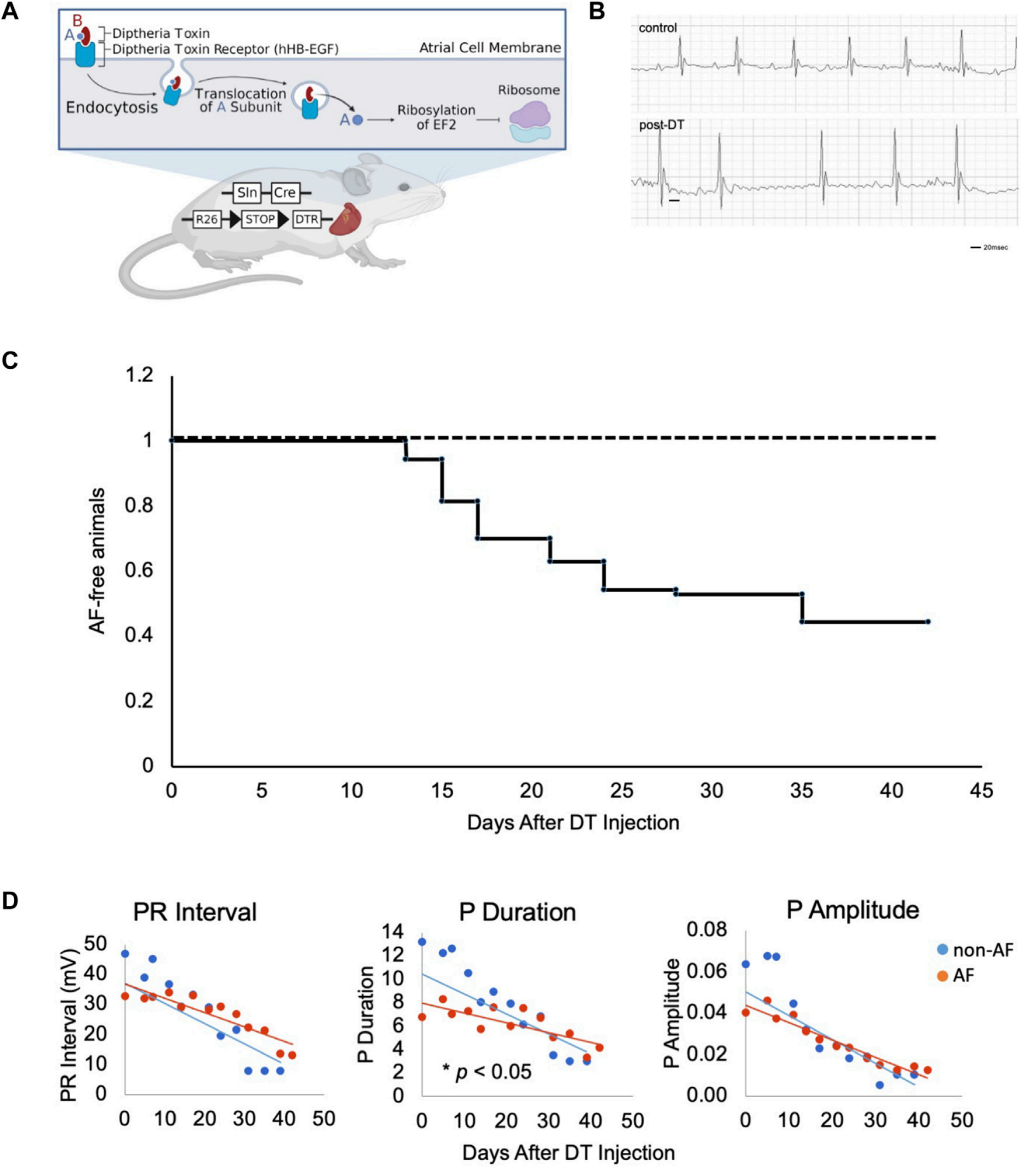
Design of murine model of atrial fibrillation **(A)**. Illustration of the generation of mouse model and experimental scheme of DT treatment. DT consists of subunits A (blue) and B (red) linked by disulfide bridges. DT binds to hHB-EGF specifically expressed on the cell surface of atrial myocytes of Sln+/Cre; R26+/DTR mice. Upon endocytosis of receptor-ligand complex, A subunit translocates into the cytosol, ribosylates host EF2, and inactivates protein synthesis, resulting in mild damage in atrial muscle. Figure drawn using BioRender software. **(B)**. Representative ECGs of two control mice at Day 18 and 21 and three AF mice at Day 17, 21 and 21 post-DT, respectively. **(C)**. Kaplan-Meyer curve of animals free from AF after DT injection. DT was injected at Day 0 and 14. (*n* = 70)

AF mouse model was generated by crossing the atrial-specific Sarcolipin (Sln)-Cre knock-in mouse (*Sln* ^*+/Cre*^) with the inducible diphtheria toxin (iDTR) mouse (*R26* ^*+/DTR*^) (Buch et al., 2005; Nakano et al., 2011) (Figure 1A). The investigation conformed to the Guide for the Care and Use of Laboratory Animals published by the US National Institute of Health (NIH Publication No. 85-23, revised 1996). All animal protocols, experiments, and housing were approved by the University of California Los Angeles institutional review committee. The study is reported in accordance with ARRIVE guidelines.

### Surface electrocardiogram and telemetry

Electrocardiograms (ECGs) were obtained under nasal isoflurane anesthesia by inserting two platinum (Pt) needle electrodes (Grass Technologies, West Warwick, RI) under the skin in the lead II configuration. The ECG data were amplified (Grass Technologies) and then digitized for analysis with HEM V4.2 software (Notocord Systems, Croissy sur Seine, France). In 6 out of 36 animals that did not develop AF at base line, the ECG data were recorded after sequential 3–50 ng/g dose intraperitoneal injections of dobutamine. For telemetry recording, an ECG telemetry sensor (ETA- F20 transmitter from Data Science Instrument) was inserted in the abdominal peritoneal cavity with leads anchored intramuscularly by a suture 2 days before the induction of AF. ECG signals were recorded at a duration and frequency of 1 min for every hour for 14 days in a single caged mouse over a radio-receiving plate under normal light-cycle and water/food accessibility.

### Histology

The hearts were fixed in 4% paraformaldehyde overnight. Fixed hearts were washed with phosphate-buffered saline (PBS), dehydrated with ethanol, ethanol + xylene, and embedded in paraffin. The sample blocks were sectioned at 4 μm thickness. Masson’s trichrome staining was performed according to the standard protocol. Fibrotic area was quantified using ImageJ software on every 12 section throughout the atria. Percent fibrotic area was calculated as the total area of fibrosis divided by the total area of atrial tissue.

### Amiodarone treatment

Eleven mice that showed AF were intraperitoneally injected with amiodarone at the dose of 100 mg/kg body weight under ECG monitoring. The treatment was deemed effective if the ECG reverted to normal sinus rhythm in 10 min after amiodarone injection.

## Results

DT binds to its receptor, human heparin-binding epidermal growth factor (HB-EGF), and is internalized to the cytoplasm where it releases its C domain, which, in turn, inhibits elongation factor-2 (EF2) and arrests peptide synthesis in target cells, causing non-specific cell damage (Figure 1A, created with BioRender). This reaction is not an immune response to specific antigen. In fact, injection of DT does not affect the wild type mice as the endogenous murine HB-EGF does not bind DT.

To selectively sensitize atrial muscle to DT, we crossed the atrial-specific Sarcolipin (Sln)-Cre knock-in mouse (*Sln* ^*+/Cre*^) with the iDTR mouse (*R26* ^*+/DTR*^) (Buch et al., 2005; Nakano et al., 2011). Sln-Cre mouse line targets Cre expression in working atrial muscle as well as the sinoatrial node (SAN) and atrioventricular node (AVN) in a highly sensitive manner (Nakashima et al., 2014). The iDTR line expresses human HB-EGF in a Cre recombination dependent manner. Thus, in the *Sln* ^*+/Cre*^ *; R26* ^*+/DTR*^ mice, atrial muscle becomes susceptible to DT treatment (Figure 1A). The *Sln* ^*+/Cre*^ *; R26* ^*+/DTR*^ mice without DT and wild-type mice with DT injection were both phenotypically normal and fertile (hereafter, “controls”). Control animals underwent a similar vehicle injection and a similar monitoring as in the animals that have developed AF, but no AF were identified in controls throughout this study. Upon intraperitoneal (i.p.) injection of low-dose DT, the *Sln* ^*+/Cre*^ *; R26* ^*+/DTR*^ mice showed irregularly irregular QRS patterns and disappearance of P waves on ECG (Figure 1B). Prolonged PR intervals and bundle branch blocks were also occasionally observed. In the C57BL6 mouse strain, the dose of DT was optimal at 0.15 ng/g i.p. at Day 0 followed by additional 0.1 ng/g i.p. at Day 14. Time course studies revealed that *Sln* ^*+/Cre*^ *; R26* ^*+/DTR*^ mice began developing AF 1 week after DT i.p. injection, and 51.4% (36/70) of mice developed AF in 1 month (Figure 1C).

**FIGURE 2:**
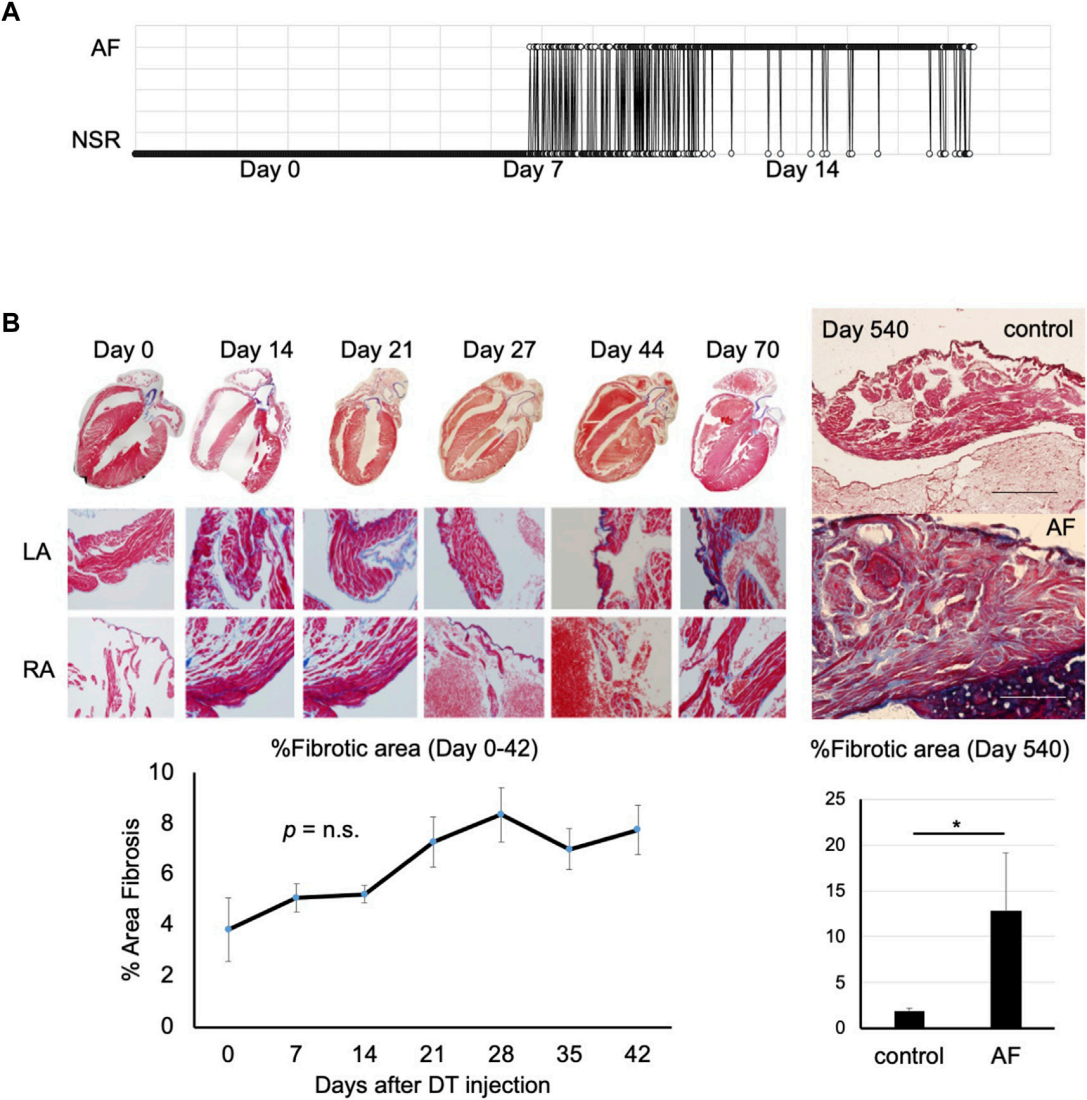
Progression of atrial fibrillation after induction **(A)**. Representative telemetry ECG indicating time in AF versus normal sinus rhythm (*n* = 4). DT was injected at Day 0. This mouse started to show paroxysmal AF at Day 8. The duration of AF gradually prolonged over time. NSR, normal sinus rhythm. **(B)**. Masson’s trichrome staining of the atrial tissue of control (left) and AF mouse (right) at Day 0 (pre-DT) to Day 540 post-DT injection. Note severe fibrosis in AF mouse at Day 540. Scale bar = 500 μm. Right panel shows the quantification of fibrotic area (*n* = 3, each; *p < 0.05).

To characterize this novel murine model of AF, telemetry analysis was performed. A telemetry chip was implanted 2 days before the induction of AF and the ECG was recorded for 1 min every hour for 14 days or until the battery ran out. A gradual shift from normal sinus rhythm to paroxysmal and persistent AF was observed over a few weeks (Figure 2A). Histological analysis revealed severe fibrosis in the atrial myocardium at the chronic phase (Day 540 post-DT injection; Figure 2B). However, the level of fibrosis was not drastically increased during the acute/subacute phases (between Day 0 and 70 post-DT injection) compared to the controls, suggesting that the severe fibrosis at day 540 post-DT was not due to the acute cardiomyocyte injury by DT but rather the consequence of long-term AF in this model. This result suggests that AF can be the cause, not only the consequence, of atrial fibrosis.

**FIGURE 3:**
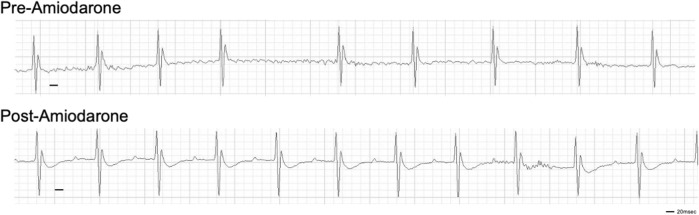
Cardioversion by i. p. injection of amiodarone. Amiodarone was injected at Day 28 post-DT injection. Representative of 9 out of 11 mice injected with DT that were successfully reverted to sinus rhythm.

Finally, to validate this mouse model as a potential tool for evaluating the efficacy of therapies, an established anti-arrhythmic drug was tested. Amiodarone (Vaughan-Williams classification class III) is the most commonly used anti-arrhythmic drug for AF. Bolus injection of Amiodarone is often used for pharmacological cardioversion of recent-onset AF with 40%–70% efficacy. Upon induction of AF following DT treatment, bolus i. p. injection of 100 mg/kg body weight amiodarone reverted the irregularly irregular rhythm to sinus rhythm within 10 min in 9 out of 11 mice tested (Figure 3). The efficacy of an established drug further suggests that this new animal model closely mimics clinical features of AF.

## Discussion

Like many other human diseases, it is evident that AF is caused by the interactions of complex genetic and non-genetic factors. Our results indicate that injury to atrial myocytes without susceptibility gene mutations is sufficient to induce sustained AF. Currently available mouse AF models include loss-/gain-of-function of signaling molecules (Verheule, 2004; Xiao et al., 2004; Davies et al., 2014; Li, 2018), metabolic (Kondo, 2018; Jin et al., 2019; Polina et al., 2020) and mechanical (Wang, 2018; Matsushita et al., 2019) insults, and mutations in susceptibility genes and ion channels (Temple et al., 2005; Zhang et al., 2019). Most mouse models of AF available to date require programmed electrical stimulation for AF induction. With the advantage of temporal specificity, no required pacing, and robustness in its phenotypic manifestation, this new mouse model can serve as a valuable tool for investigating the underlying mechanisms in high resolution along the time course of AF disease progression. In conjunction with the plethora of genetically-modified mouse lines with susceptibility gene mutations, this mouse line can also be utilized for the analysis of the interaction between genetic and non-genetic factors in the pathogenesis of AF. For example, it would be interesting to investigate if atrial muscle pathway injury would have been revealed *via* proteomics. Finally, the scalability and robustness of this mouse model will also be advantageous for testing new drugs and new ablation procedures for AF including antifibrotic drugs and autonomic nervous modulation therapies. Thus, this new mouse model can serve as a valuable platform for understanding disease mechanisms and developing new therapeutics for AF.

## Data Availability

The original contributions presented in the study are included in the article/Supplementary Materials, further inquiries can be directed to the corresponding author.
